# Clinical characteristics of patients seeking medical advice for nasal symptoms in Bulgaria with special focus on children

**DOI:** 10.1186/s40413-016-0103-6

**Published:** 2016-04-04

**Authors:** Tihomir B. Mustakov, Todor A. Popov, Tanya Z. Kralimarkova, Maria T. Staevska, Vasil D. Dimitrov

**Affiliations:** Clinic of Allergy and Asthma, Medical University, 1, Georgi Sofiyski St., 1431 Sofia, Bulgaria

**Keywords:** Allergic rhinitis, Asthma, Children, Nasal symptoms, Treatment practices

## Abstract

**Background:**

In an attempt to circumvent low response rates and high cost of classical epidemiological trials, we carried out a real-life survey among practicing physicians consulting patients for nasal symptoms. In this fragment of our work we analyze similarities and differences between children and adults and within the different strata of pediatric age.

**Methods:**

A survey was carried out by 69 physicians across Bulgaria (general practitioners, allergists and otorhinolaryngologists) and made possible calculation of the proportion of subjects with nasal symptoms from all other patients seen. Its structure allowed classification of rhinitis according the ARIA guidelines.

**Results:**

Out of the 1685 completed survey forms, 506 pertained to the age group below 18 years. The gender predominance differed in children and adults: 57.3 % vs. 42.8 % of males respectively, *P* < 0.001. The prevalence of persistent rhinitis in children was 55.7 %, lower than in adults, 63.3 %, *P* = 0.004. In both pediatric and adult patients moderately severe and severe forms of rhinitis prevailed, 93.7 % vs. 94.6 %, with nasal obstruction as leading symptom: 59.9 % vs. 58.8 %. Cough was significantly more prevalent among children, 72.5 %, gradually decreasing until reaching adulthood, 58.7 %, *P* < 0.001. Prevalence of doctor diagnosed asthma was also higher among children, 25.1 %, than in adults, 19.5 %, *P* = 0.011. A gradient for characteristics, which were different in children, emerged across the pediatric age strata.

**Discussion:**

Our study uses an unorthodox design targeting the patient population visiting physicians’ offices because of nasal symptoms, achieving a much higher level of credibility of the results at minimal expense. As we base our survey on international guidelines, we believe this approach demonstrates the applicability of such consensus documents for practical purposes when in the hands of qualified physicians.

**Conclusions:**

Moderate and severe rhinitis symptoms motivate patients and their guardians to seek medical advice. While nasal congestion is a leading bothersome symptom in both adults and children, specific other features characterize the pediatric age and differ across its strata.

## Background

Epidemiological studies are the starting point in clinical medicine. The credibility of the obtained results and inferences, however, depends to a great extent on achieving adequate response rates. Over the years we carried out several epidemiological studies in Bulgaria using study designs involving postal surveys with self-administered questionnaires or the services of non-medical interviewers, but the yielded response rates were quite low and did not allow publishing of the results. For this reason we decided to involve practicing physicians when designing the Symptoms of Nasal Inconvenience Fact Finding (SNIFF) project to study some practical aspect of the management of allergic rhinitis [1]. It involved a survey, which assessed the incidence of visits to physicians’ offices due to nasal complaints and classified the patients actively seeking medical advice according the severity and persistence of their symptoms following the Allergic Rhinitis and its Impact on Asthma (ARIA) guidelines. Unlike standard epidemiologic studies which target the general population by means of interviewer- or self-administered questionnaires, the SNIFF project was based on the real-life setting of outpatient practices. The collection of data followed a standardized pattern and was documented by physicians who had more or less specialized experience and training and were committed to rendering professional help to patients visiting their offices. The finding that about 14 out of 100 patients visiting general practitioners and specialists did so because of nasal symptoms provided an estimate of the burden, which nasal pathology in general and rhinitis in particular pose on the national health care system. This segment of the patient population is of practical interest, as it represents those subjects who consider their complaints bothersome enough to warrant the time and expense involved in consulting a physician. In the paper presenting the overall results of the SNIFF project we established that these would be the patients with moderate to severe rhinitis and those who rank nasal congestion as a leading symptom [1]. Now we analyze the results with particular focus on the pediatric patients.

The pediatric population is rather inhomogeneous as it presents a gradient from total unawareness of disease symptoms in early infancy, when parents need to take decisions about health issues of their children, to full perception of the discomfort due to pathological symptoms with the advent of adolescence. Based on the level of dependence on parental care, three pediatric age periods are generally accepted: below 6 years, between 6 and 12 years and teen-age/adolescence. Thus, in the age below 6 years health related decisions including the need to see a doctor are taken by the parents, while later on the pathologic symptoms are recognized and reported increasingly by the growing individuals. As a consequence, the decision to set up medical appointments is shifted from the sole responsibility of the parents to decisions taken following a conscious dialogue between children and their parents/guardians. In Bulgaria however most parents do not consider their child ill if it is not febrile or have unstoppable cough or severe pain. For this reason the children, particularly in preschool age have months even years of delay before referring to medical specialist, despite the presence of nasal, bronchial and allergic pathology. This circumstance has to be taken into consideration when collecting epidemiological data for the pediatric age, as parental awareness and sensitivity for health issues may bias the results one way or another.

Allergic rhinitis is of particular interest in the early individual development as it is considered part of the atopic trait and risk factor for the development of asthma. Attempts to assess the prevalence of rhinitis in Bulgarian children through classical epidemiological approaches have been inconclusive because of organizational and cultural problems [2]. The information from our participation in the ISAAC phase 3 project indicated 24 % of the 12–13 year old children had allergic rhinitis related symptoms [3]. Studies in other countries find higher prevalence of allergic rhinitis symptoms [4–7]. This information, however, reflects the general pediatric population. Identifying the mild forms of allergic rhinitis through surveys of the general population may be difficult, as the borderline between “normal” and “pathological” is rather blurred and subject to individual lay judgment.

The aim of the SNIFF project was to estimate the prevalence of nasal symptoms in both pediatric and adult outpatients consulting physicians because of them. The specific purpose of this paper is to analyze the data for the pediatric age group, to uncover peculiarities across this age spectrum and to seek similarities and differences with the adult age.

## Methods

### Project design

We invited randomly selected general practitioners (GP) and specialists consulting both pediatric and adult patients for nasal complaints (otorhinolaryngologists (ORL) and allergists (ALRG)) across Bulgaria to take part in this cross-sectional survey. Physicians, who participated in the study, did not receive compensation for their work. Each participating doctor received personal code, identifying him/her as GP, ORL or ALRG. For a period of 20 working days (between January and March, prior to the pollen season to minimize potential bias) they had to fill in survey forms for every patient with nasal symptoms attending their practices. Forms were not filled in for the other patients who did not visit because of without nasal symptoms, but track was kept of their overall count. The count was started from 1 at the first day of the study, including all entries in the physician’s log book preceding the first patient visiting for nasal symptoms. The number of patients without nasal symptoms was recorded till the end of the last day of the study to be able to calculate the total number of patients seen.

The study was designed to minimize expenses. It was approved by the Ethics Committee of Alexander’s University Hospital in Sofia, Bulgaria. The Committee judged that it was not necessary for the patients to sign written consent because no study specific investigations or treatments were envisaged apart from the regular work-up and no identifiable personal data were registered in the study materials.

### Survey structure

All participating physicians underwent a 2 day course on how to conduct the survey and to get familiar with the questionnaire.

Physicians filled in the survey forms only for the patients with nasal symptoms with basic demographic and disease information but no personal identity data (14 questions). A set of entries was used to document the nasal symptoms present and to rank them according to the amount of discomfort they were causing (Question #3: “Please rank the symptoms ‘stuffy nose’, ‘runny nose’, ‘itchy nose’ and ‘sneezing’ according to the level of discomfort they bring about”). This allowed classification of patients as predominantly “blockers” (with congestion as their most prominent symptom) or “runners” (with rhinorrhea as their leading symptom). Although a classification into “blockers” and “runners” is not officially accepted in the international and Bulgarian national guidelines, this distinction was registered in our study because of possible therapeutic implications on the choice of pharmacological treatment. Further entries into the survey form allotted patients into groups with intermittent or persistent rhinitis (Question #4: “How often do you have nasal symptoms: i.) ≤ 4x/week or < 4 weeks, or ii.) > 4x/week or > 4 weeks”) and allowed to assign a severity grade to their condition on the basis of impairment of nighttime sleep, daytime activities and performance at school/work (Question #5) in line with the Allergic Rhinitis and its Impact on Asthma (ARIA) guidelines [8, 9].

The questionnaire included also entries tackling the sensitivity to pollens, cough and the impairment of the sense of smell as an important functional feature of the nose (Questions 6, 7 & 9). The role of recurring infections in triggering and maintaining the symptoms of rhinitis is always open to debate. Because of this and as our survey was conducted during the winter, we included a question whether the nasal symptoms were triggered or associated with viral infections (Question #2). A question was also asked if a doctor has documented the following diagnoses: asthma, chronic sinusitis, adenoid hypertrophy, nasal polyp(s) (Question #11). Since the study was focused on nasal symptoms all other health issues were considered as comorbidities even if they were more severe than rhinitis.

The survey provided also assessment of the treatment practices, as physicians indicated their decisions on a multiple-choice list (Questions #12, 13, 14).

### Statistical analysis

Frequencies with number of cases and percentages were calculated for all dichotomous and ordinal variables. Percentages; significance was based on Pearson’s *χ*^2^ test. Prevalence differences for subgroups with alternative subtypes/severities/leading symptoms were calculated for children and adults, and separately within the stratified pediatric age groups were expressed as percentages. Comparisons were calculated using cross-tables and significance was based on *χ*^2^ test. Binary logistic regression for dichotomous variables of interest for the analysis was used to calculate odds ratios (ORs) with 95 % confidence intervals (Cls). A 2-tailed *P*-value ≤ 0.05 was considered as the cutoff for significance.

## Results

### General characteristics the pediatric and adult patient groups

Out of the 1685 completed surveys by 69 physicians (30 GPs, 39 ENTs and ALRGs) 506 belonged to the pediatric age group. The overall mean number of patients entering the physicians’ offices until a patient with rhinitis symptoms stepped in (in-between patients) was 6.4. As adults constituted the majority of rhinitis patients in our population, 1179, the number of in-between patients for per adult was 9.1, while 22.2 in-between patients preceded the visit of a pediatric patient. For the distinct age groups in the pediatric age these numbers were 69.3 (children < 6 years), 61.0 (children between 6 and 12 years) and 61.3 for adolescents. The stratification of the survey population into age groups and gender is shown on Table 1.

**Table 1:** Age strata and gender differences within the surveyed population

Age strata	Children <5 years	Children 6–12 years	Adolescents 13–17 years	All aged <18 years	Adults ≥18 years	Altogether
Number	155 (9.2 %)	176 (10.4 %)	175 (10.4 %)	506 (30.0 %)	1,179 (70.0 %)	1,685 (100.0 %)
% males (of stratum)	48.4 %	61.4 %	61.1 %	57.3 %	42.8 %	47.2 %
Significance for gender differences children/adults				*P* = 0.000		
Medium age (years)	5	9	16	9	39	30
Age range (years)	1–5	6–12	13–17	1–17	18–88	1–88
Mean age (years)	4.3	9.3	15.5	9.9	41.3	31.9

### Differences between pediatric and adult rhinitis patients

Besides showing the prevalence of the different subtypes of rhinitis and the related comorbidities, we chose to present the comparisons of between children and adults and in the pediatric subgroups of the surveyed population by means of ORs as indicated in the ‘Methods’ section (Table 2). The adults were taken as baseline comparator, so that OR values above or below 1.0 (if significant, *P* ≤ 0.05) meant increase or respectively decrease of the likelihood that children would have a feature characterizing their rhinitis. Thus children were less likely to have persistent rhinitis and impairment of their sense of smell than adults, but did not differ in terms of the severity of the disease. At the same time they were more prone to have cough and doctors’ diagnosed asthma than adults. Interestingly, congestion emerged as a leading symptom in both age groups without significant differences between them.

**Table 2:** Differences and similarities between the pediatric and adult populations

Diagnosis^#^, characteristics, comorbidities	Prevalence (%)		Odds ratio	95 % confidence interval	*P* value
	Children (*n* = 506)	Adults (*n* = 1179)			
Persistent rhinitis	55.7	63.3	0.731	0.591 ÷ 0.903	0.004
Moderately severe/severe rhinitis	93.7	94.6	0.850	0.549 ÷ 1.317	0.468
Leading congestion	59.9	58.8	0.954	0.772 ÷ 1.180	0.666
Exacerbation during the pollen season	50.2	48.2	1.087	0.882 ÷ 1.339	0.436
Impaired school/work performance	43.6	27.8	1.258	1.004 ÷ 1.576	0.046
Impaired sense of smell	19.1	34.7	0.392	0.302 ÷ 0.508	0.000
Cough	72.5	58.7	1.859	1.480 ÷ 2.336	0.000
Asthma^(a)^	25.1	19.5	1.394	1.089 ÷ 1.786	0.008
Chronic sinusitis^(a)^	19.8	22.4	1.171	0.905 ÷ 1.516	0.230
Adenoid hypertrophy^(a)^	18.6	0.4	14.618	5.370 ÷ 39.793	0.000
Nasal polyp(s)^(a)^	0.8	10.4	0.019	0.008 ÷ 0.046	0.000

^a^Children below 6 years were excluded from the analysis as the indicated variables cannot be documented reliably for this age group

^#^Data are based on doctor made diagnosis

In response to the question in our survey whether the nasal symptoms are usually triggered by and associated with infections, 58.7 % of children and their parents considered infection to be associated with their symptoms always or quite often; conversely, only 42.3 % of the adult patients attributed their bothersome nasal symptoms to infection (*P* < 0.001).

### Differences within the school age pediatric group

As school age between 6 and 17 years is the transition form childhood to maturity, we compared the two distinct age periods, 6 ÷ 12 vs. 13 ÷ 17 years, to see whether they can identify any gradient in the ontogenetic development (Table 3). The significantly higher incidence of cough and asthma in the younger group may reflect the immaturity of respiratory system and the higher number of infections in this age group. The decreased incidence of persistent symptoms in second age group may be due to the increased pollen sensitization in youngsters. The percentage of cases where infection was indicated as the most likely trigger of nasal symptoms was highest in children < 6 years, 83.5 %, vs. 52.8 (children 6–12 years) and 51.4 %, *P* < 0.001.

**Table 3:** Differences within the school age groups of pediatric patients

Diagnosis, characteristics, comorbidities	Prevalence (%)		Odds ratio	95 % confidence interval	*P* value
	Children 6–12 years (*n* = 176)	Adolescents 13–17 years (*n* = 175)			
Persistent rhinitis	60.8	50.9	1.498	0.981 ÷ 2.289	0.061
Moderately severe/severe rhinitis	96.0	92.6	1.937	0.754 ÷ 4.978	0.170
Leading congestion	62.5	56.6	0.782	0.510 ÷ 1.198	0. 258
Exacerbation during the pollen season	50.6	57.1	0.776	0.509 ÷ 1.183	0.238
Impaired school/work performance	42.6	43.6	0.933	0.611 ÷ 1.423	0.746
Impaired sense of smell	16.5	21.7	0.716	0.418 ÷ 1.225	0.222
Cough	73.9	58.3	2.115	1.341 ÷ 3.333	0.001
Asthma^(#)^	31.2	20.6	1.759	1.081 ÷ 2.862	0.023
Chronic sinusitis^(#)^	19.9	22.3	1.155	0.691 ÷ 1.931	0.582
Adenoid hypertrophy^(#)^	15.3	2.9	0.162	0.061 ÷ 0.432	0.000
Nasal polyp(s)^(#)^	0.0	2.3	-	-	-

^#^Data are based on doctor made diagnosis

### Treatment

Significant differences emerged between children and adults on one hand and within the pediatric age group when prescribed treatments were compared (Table 4).

**Table 4:** Treatment differences across the pediatric age strata and between children and adults

Age strata patients	Children <5 years	Children 6–12 years	Adolescents 13–17 years	All aged <18 years	Adults ≥18 years	Altogether
% on oral H1-blockers	51.6 %	54.0 %	44.6 %	50.0 %	50.5 %	50.3 %
Significance	*P* = 0.188			*P* = 0.861		
% on nasal steroids	36.1 %	55.7 %	49.7 %	47.6 %	61.2 %	57.1 %
Significance	*P* = 0.001			*P* = 0.000		
^(a)^% on decongestants	0.0 %	0.0 %	0.6 %	0.2 %	1.5 %	1.1 %
Significance	*P* = 0.114			*P* = 0.020		
% immuno-therapy	8.4 %	17.0 %	20.6 %	15.6 %	9.8 %	11.5 %
Significance	*P* = 0.008			*P* = 0.001		

^(a)^Decongestant were applied mainly orally as part of drug combinations

As in the previous paper dedicated to the SNIFF project, we analyzed differences between prescription practices of GPs and specialists, ENTs and ALRGs. Conceivably, as ALRGs exclusively deal with immunotherapy, they differed from ENTs and GPs in prescribing this particular treatment. However, no other differences specifically associated with prescription practices in the pediatric age appear, we refrain to present them in this paper.

## Discussion

In designing the SNIFF project we chose to accept that the mild segment of allergic rhinitis morbidity would be underscored, as the mild cases were much less likely to seek professional medical advice. The assessment of the proportion of mild rhinitis cases would have required a classical epidemiological approach involving much more resources and substantial bias. Thus, an important achievement of our study was that it did not rely on the response rate from the general public, but made use of the conscientious voluntary contribution of physicians. This approach circumvents the pitfalls associated with poor response rates or bias associated with postal or Internet based questionnaires or the low quality of data collected by non-medical field workers. We reasoned that much more accurate information could be derived by surveying practices of physicians consulting patients from all layers of the general population, thus characterizing the patients with nasal symptoms serious enough to prompt medical consultation. To our knowledge we are the first to use such a real life based design [10–12]. The benefit of using data collected by physicians can have important implications on administrative level. Within the context of this article, it can also help address peculiarities of the pediatric age in elaborating national guidelines.

Stratifying those patients according to age made possible getting specific insight about differences between adults and children in terms of duration and severity of rhinitis symptoms. There might be some doubts that the data concerning children might be confounded by the personal input of their parents/guardians, but the way the results turned up make good clinical and epidemiological sense. Thus, congestion appeared as the main motive to seek medical advice for both children and adults, which was in line with the findings of the large majority of epidemiological studies and the international and national guidelines [13–15]. Logically, it was the moderate-severe and severe forms of rhinitis that equally motivated the visit to the physicians’ offices. However, distinct differences emerged, which could be regarded as signs of chronicity evolving over time in the adult population. The significantly higher prevalence of persistent rhinitis in the adult age can be one such indicator. The impairment of the sense of smell was another much more pronounced signal captured in the adults. On the other hand, children were much more prone to cough, which could be explained with the more sensitive cough reflex [16, 17]. This was confirmed with the analysis of the pediatric strata, where cough was preponderant in the younger children as seen in Table 3. With the advent of adolescence cough and airway responsiveness seemed to subside. Thus the common knowledge about the higher prevalence of childhood asthma also found support in our study.

In the preceding paper on the SNIFF project, we uncovered differences between the type and severity of rhinitis in 1685 patients with nasal symptoms as seen by GPs, ENTs and ALRGs. The similar analysis of the 506 pediatric cases, though, did not reveal significant differences.

Interestingly, there were gender differences between adults and children, which expectedly pointed towards increased rhinitis symptoms in adult women. This is in line with other general epidemiological data [18, 19]. The gender data from the pediatric strata are less straight forward: a larger pediatric sample might have given a better outline of gender differences in the pediatric age. Similarly, differences in drug prescription for children and adults would have allowed more certainty if a larger pediatric sample was involved [20–23].

We found significantly higher percent of immunotherapy in pediatric patients which reflects the specific practice in our country and the fact that children are seen by allergists more often than adults.

Despite the fact that the study was done in Bulgaria, we believe this information would be of interest to a much wider international audience, as we used an unorthodox design targeting the patient population visiting physicians’ offices because of nasal symptoms, achieving a much higher level of credibility of the results at minimal expense. As we base our survey on international guidelines, we believe this approach demonstrates the applicability of such consensus documents for practical purposes when in the hands of qualified physicians.

Our study has certainly limitations, some of which could have to do with the limited budget. Certainly, a larger pediatric sample might have rendered the comparison with the adult population more reliable and convincing. Among our specialists we did not have pediatricians, which might have affected the results for our pediatric population and especially the lowest age stratum. However, the intent was to involve physicians who would be seeing both pediatric and adult patients. In conducting the survey we adhered to the standard referral practices of the Bulgarian health-care system.

## Conclusion

In conclusion, symptoms of persistent rhinitis motivate children and their parents to seek medical advice. While nasal congestion of sufficient severity is a leading bothersome symptom in both adults and children, specific other features like cough sensitivity, sense of smell impairment and comorbidities characterize the pediatric age. Furthermore, rhinitis symptoms in adults may also reflect the developments of chronic pathological changes in the underlying nasal tissues, while maturing of the immune system and the local defenses of the mucosa maybe beneficial for the evolution of pediatric morbidities.
